# Sustainable successes in third-party food delivery operations in the digital platform era

**DOI:** 10.1007/s10479-023-05266-w

**Published:** 2023-04-12

**Authors:** Hau-Ling Chan, Ting-Ting Cheung, Tsan-Ming Choi, Jiuh-Biing Sheu

**Affiliations:** 1 Department of Logistics and Maritime Studies, Faculty of Business, The Hong Kong Polytechnic University, Hung Hom, Hong Kong; 2 Division of Business and Hospitality Management, College of Professional and Continuing Education, The Hong Kong Polytechnic University, 9 Hoi Ting Road, Yau Ma Tei, Kowloon, Hong Kong; 3 Centre for Supply Chain Research, University of Liverpool Management School, Chatham Building, Liverpool, L69 7ZH UK; 4 Department and Graduate Institute of Business Administration, College of Management, National Taiwan University, Roosevelt Road, Taipei, 10617 Taiwan, Republic of China; 5 Department of Business Administration, National Taiwan University, Taipei, Taiwan, Republic of China

**Keywords:** Third-party food delivery, Digital platform, Future research agenda, Sustainability

## Abstract

In the digital era, third-party food delivery operations are very popular all around the world. However, to achieve a sustainable operation for food delivery businesses is a challenging issue. Motivated by the fact that there is a lack of consolidated view towards the topic in the literature, we conduct a systematic literature review to identify how to achieve a sustainable operation for third-party food delivery and highlight the recent advances in this important area with the discussion of real-world practices. In this study, first, we review the relevant literature and apply the triple bottom line (TBL) framework to classify prior studies into economic sustainability, social sustainability, environmental sustainability, and multi-dimensional sustainability. We then identify three major research gaps, including inadequate investigation on the restaurant’s preferences and decisions, superficial understanding on the environmental performance, and limited examination on the multi-dimensional sustainability in the third-party food delivery operations. Finally, based on the reviewed literature and observed industrial practices, we propose five future areas that deserve an in-depth further investigation. They are namely applications of digital technologies, behaviors and decisions of the restaurants, risk management, TBL, and post-coronavirus pandemic.

## Introduction

### Background

Due to the coronavirus pandemic, governments all around the world have encouraged the provision of food delivery services or implemented different dine-in restrictions. For example, the UK government advised the restaurants to sell food online and provide delivery service.^1^ In Hong Kong, the dine-in service was prohibited after 6 pm to avoid the spread of virus (Kathleen & Lo, 2022). With a growing necessity for stay-at-home during the coronavirus pandemic, restaurants must adopt new distribution channels for delivering their foods to customers. Hence, third-party food delivery platforms such as Foodpanda and Deliveroo consolidate a trend of significant growth (Yang et al., 2020).

According to Pandey et al. (2022), food delivery platforms can be categorized into two types: (i) Restaurants operated, and (ii) third-party food delivery platform operated. For the latter one, restaurants form a partnership with third-party food delivery platforms who provide the food delivery services to customers (Preetha and Iswarya, 2019). Under such operations, a customer first places an order through a third-party food delivery platform (e.g., website or application (app)), and the ordering details will then be passed to the corresponding restaurants. Later on, the third-party food delivery platform will arrange personnel (i.e., rider) to pick up the food from the restaurants and provide delivery services to the customer.^2^ The revenue of the third-party food delivery platforms comes from both restaurants and customers with five major sources (Ahuja et al., 2021). To be specific, restaurants have to pay the commission fees. It was reported that the third-party food delivery platform charges around 20 to 25 percent of each order from those exclusive restaurants, 30 to 35 percent of each order from non-exclusive restaurants, and 3 to 8 percent from each customer self-pickup order as restaurant commission fees (Leung, 2022). In case the restaurants desire to promote their brands through the third-party food delivery platform (e.g., website or app), they will be charged for the advertising services. On the other hand, customers are required to pay the delivery fees and service fees. For the delivery fees, it depends on traveling distance and time between the restaurants and customers. Apart from delivery, the third-party food delivery platform also provides various services, such as 24/7 ordering, pre-ordering, customer rating on the restaurants (Lichtenstein, 2020), which are regarded as the service fees. Finally, customers can tip the riders which may lower the operating cost of the food delivery platform for staff retention. It is found that different food delivery platforms (e.g., Doordash, Uber Eats) have different settings on the fees charged to both restaurants and customers (Lichtenstein, 2020). Overall, in a typical third-party food delivery system, it involves the participation of the restaurants, third-party food delivery platforms, riders, and end-customers, and they are connected by the digital technologies, and the operations are driven by big data analytics (Bozkaya et al., 2022).

Overall, with the support of information communication technology and the governments’ restrictions on the dine-in services, there is a significant business growth of the third-party food delivery industry during the coronavirus pandemic (Chokshi, 2020). It is estimated that the market of food delivery industry has grown four to seven times in 2020 and 2021, compared with 2018 (Ahuja et al., 2021), and the market revenue of the third-party food delivery services will hit the level of US$208 billion in 2022 and reach US$302.5 billion by 2027.^3^

In addition to the economic performance, social and environmental sustainability aspects are also crucial in constructing a sustainable economy (Song et al., 2022). It is reported that about 45% of the unwanted materials sending to the U.S. landfill is in fact food waste and food packaging,^4^ and the carbon emissions generated from the food delivery service will increase about 32% by 2022 (Joselow, 2020). Furthermore, more than half of the delivery riders suffered from work injuries and 60% of them claimed that they were not paid after injuries (Young, 2021). To sustain a long-term success, food delivery companies should contribute to the economic, social and environmental sustainability to improve both customers’ short-term values and all stakeholders’ long-term well-being (Barthel & Ivanaj, 2007; Crane & Desmond, 2002). Theoretically, the triple bottom line (TBL) framework addresses that business firms should commit to the economic, social and environmental responsibilities. By applying the TBL framework, businesses can improve their financial performance, satisfy their stakeholders and develop competitive advantages to differentiate them from the rivals in a dynamic market (Schulz & Flanigan, 2016). Therefore, in this study, the TBL framework is adopted to let us understand the prior studies in each sustainability aspect which will provide a guideline for developing the future research agenda for the food delivery operations.

### Contribution and organization

Third-party food delivery operation is a timely topic especially during the coronavirus pandemic. However, there is a lack of consolidated and up-to-date view of this topic, which will be crucial for the post-pandemic era. This study hence aims to fill this gap by conducting a systematic literature review to identify how to achieve a sustainable operation for third-party food delivery. As a remark, in the existing literature, Seghezzi et al. (2021) also conducted a comprehensive literature review on the food delivery services and identified the role of each player in the food delivery operations. Different from Seghezzi et al. (2021), this study not only provides an up-to-date view towards food delivery operations with the application of the triple bottom line (TBL) framework, but also highlights the recent advances in this important area with the discussion of real-world practices. Besides, this study also proposes future research directions to enriching the knowledge in this type of businesses during and after the coronavirus pandemic.

The rest of this paper is organized as follows. In Sect. 2, we present the methodology in selecting the relevant articles on the sustainable third-party food delivery operations and illustrate the distribution statistics results. In Sect. 3, we review the selected articles based on economic sustainability, social sustainability, environmental sustainability, and multi-dimensional sustainability. In Sect. 4, we discuss the findings and propose future research agenda on five different areas, namely, digital technologies in third-party food delivery operations, behaviors and decisions of the restaurants, risk management, TBL, and post-coronavirus pandemic. Finally, we present the concluding remarks in Sect. 5.

## Methodology and distribution statistics

This study presents a review of third-party food delivery operations and its sustainable successes in the digital platforms era by addressing the economic, social and environmental sustainability under the triple bottom line (TBL) framework.

**Fig. 1:**
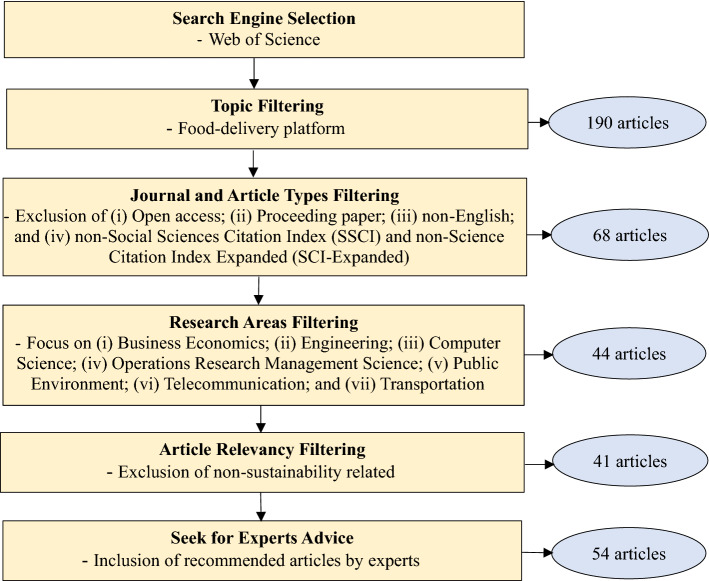
Articles selection process adopted in this study

To collect the relevant articles for review, a systematic search procedure was adopted. First, Web of Science database was used for articles identification. Next, we searched the articles by using food-delivery platform as the topic and obtained 190 articles. As we focused on those articles published in well-established journals, we performed several rounds of articles filtering. To be specific, we excluded open access journals in the quick filters function, proceeding papers in the document types of classification, non-English articles in the languages, but selected both Social Sciences Citation Index (SSCI) and Science Citation Index Expanded (SCI-Expanded) journals. In this stage, we had 68 articles. Besides, we further refined the articles by considering the research areas in business economics, engineering, computer science, operations research and management science, public environment, telecommunication, and transportation which resulted in 44 articles. We then read the abstract and introduction section of each article and determined its relevancy to address the sustainability issue. We found that three articles were not relevant and should be excluded. Finally, we sought for article recommendation from scholars studying the third-party food delivery operations.^5^ Eventually, we reviewed 54 articles. The article selection process was conducted in August 2022. Figure 1 illustrates the articles selection process adopted in this study.

**Fig. 2:**
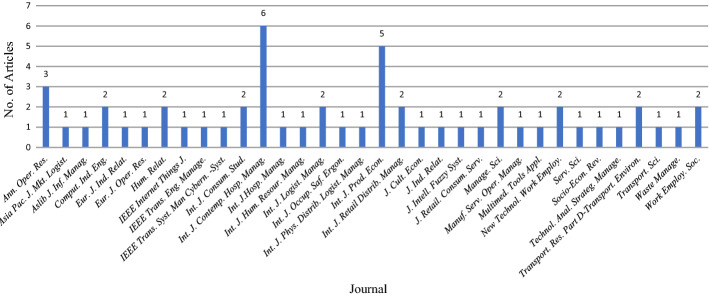
Specific journals and the publication numbers of the reviewed articles

**Fig. 3:**
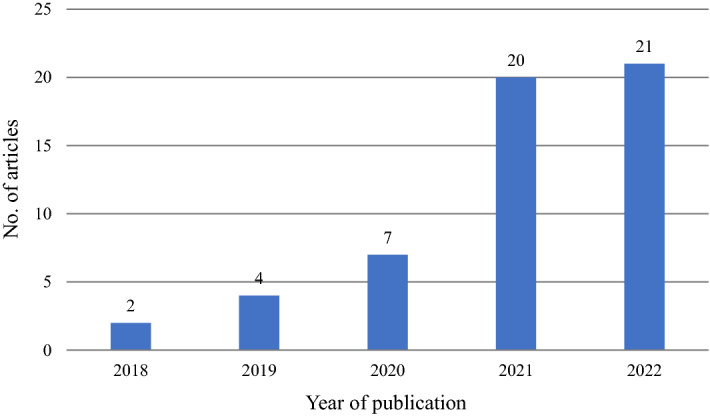
The total number of publications in each year

**Fig. 4:**
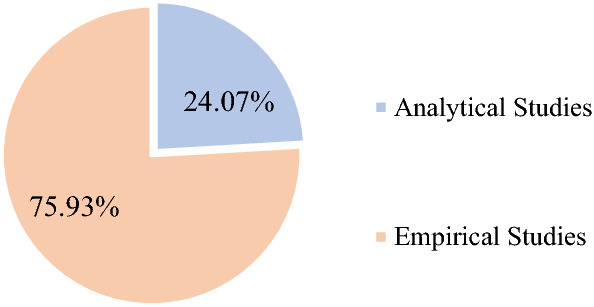
Distribution of the reviewed articles by research types

After collecting the articles, descriptive analyses were conducted. Figure 2 summarizes the journal publications of the reviewed articles. In this study, the relevant articles were published in 34 journals, including Annals of Operations Research, International Journal of Production Economics, Management Science, Manufacturing & Service Operations Management, etc. Figure 3 shows the number of publications in each year. It is obvious that the publication number has increased exponentially from 2018. As a remark, the number of publications in 2022 was only counted from January 2022 to August 2022, and hence the whole year figure should be much higher. Finally, Fig. 4 presents the distribution of the articles based on analytical and empirical studies. In this paper, “empirical” studies refer to those which employ empirical data in the analyses and cover the ones with computational experiments, qualitative case studies, and statistical based quantitative studies. On the other hand, “analytical” studies mainly represent those which are based on mathematical models with theoretical results derived in closed-form. The majority of the articles belongs to the empirical studies in which one of them is a review type of research article.

## Literature review

In this section, we review the relevant articles based on the triple bottle line (TBL) framework, i.e., economic, social and environmental sustainability. Høgevold et al. (2014) indicated that an organization needs to address economic development continuously, while it is also important to address both social and environmental impacts. The prevalence of food delivery business is caused by the coronavirus pandemic in recent times, and its sustainable development should be carefully examined to ensure that the third-party food delivery businesses achieve sustainability by maximizing the positive impacts and minimizing the negative impacts (Li et al., 2020). In the following, we classify the relevant articles into economic sustainability, social sustainability, environmental sustainability, and multi-dimensional sustainability for review.

### Economic sustainability

Economic sustainability addresses the financial performance of an organization for survival (Jawahar et al., 2017). Therefore, in this study, economic sustainability refers to the financial performance of food delivery platforms and restaurants. We find that the existing literature related to economic sustainability in food delivery operations has considered consumer behaviors and various operational problems. We review the relevant studies as follows.

#### Consumer behaviors and preferences

One important issue that will significantly affect the financial performance of both food delivery platforms and restaurants is the customer relationship management as it can build a higher customer loyalty and generate a repeated purchase intention in the future. Jha (2022) proposed techniques (Generalized Savitzky-Golay Filter (GS-GF) and Hybrid Self Constructing Neural Fuzzy based African Buffalo Optimization (HSCFN-ABO)) to extract features of customer comments and classified them into food quality, delivery and payment categories to improve customer relationship management. In addition, consumer behavior of using the food delivery services is also one prominent research area. To be specific, Gunden et al. (2020) statistically investigated the factors affecting customer intention to adopt online food delivery services. They found that having a higher level of performance expectancy, congruity with self-image, habit and mindfulness can boost the customer’s intentions to adopt the online food delivery services. However, customer’s impulse purchase tendency will generate a negative effect on it. Similar to Gunden et al. (2020), Francioni et al. (2022) also examined the customer’s intention to use food delivery platforms. Differently, Francioni et al. (2022) considered another set of factors that may affect the customer’s intention and investigated the moderating role of gender. Their statistical results showed that perceived healthiness, perceived hygiene, quarantine procedures, perceived ease of app use, and attitude toward the benefits of using food delivery services are the determinants positively associated with the customer’s intention to utilize the online food delivery services continuously. By considering the moderating effect of gender, the perceived healthiness is a crucial factor for both male and female customers to use the online food delivery service. Wang and He (2021) explored the effects of ease of food access and built environment on the on-demand food delivery (ODFD) services by using published data. They revealed that both issues will affect the ODFD services consumption. The proportion of using the land for green spaces and the degree of urbanization have negative and positive effects on using the ODFD services, respectively. Yen (2022) statistically explored the relationship between channel integration and food delivery platform (FDP) services consumption by considering different perceived values and perceived risks, and then examined how the customer’s innovativeness and experience moderate such relationships. It was found that channel integration through perceived usefulness, enjoyment and price will affect FDP services consumption. In addition, customer’s innovativeness and experience demonstrate a moderating effect in such relationship.

Food delivery operations are supported by apps and there are relevant articles studying consumer behavior of using the food delivery apps. Kapoor and Vij (2018) statistically explored how the attributes of the mobile apps affect customer intention to use the online food ordering services. They concluded that the mobile apps attributes of collaboration designs, such as promotion and discount offerings, have the greatest effect on the online food ordering services utilization, followed by information design, navigational design, and visual design. Kaur et al. (2021) identified the values of stimulating the food-delivery app usage. They showed that visibility has the greatest impacts on food delivery apps usage intention, followed by affordances, values for money and prestige social benefits. However, food safety and health concerns are not significantly associated with the apps’ usage intention. Yeo et al. (2021) statistically evaluated the driving forces of customer’s repurchase intention of the food delivery apps. They found that perceived usefulness, social influence and trust are the drivers of repurchase intention of the food delivery apps. However, effort expectancy, information quality and perceived risks are not significantly associated with the repurchase intention. Raza et al. (2022) statistically explored the trust transfer from the online food delivery apps to the restaurant and its relationship with the reuse intention of the apps. They presented that trust disposition and online ratings will affect customer’s trust in the food delivery apps which will eventually generate a positive effect on the customer’s trust in restaurant and apps reuse intension. Besides, the perceived effectiveness of dispute resolution acts as a moderator between the trust in an app and the trust in a restaurant.

Some other studies have examined brand equity of the food delivery platforms and evaluated customer selection preference. For instance, Ahn and Kwon (2021) discovered that perceived economic exchange, social exchange and mutual interests with the food delivery apps have positive relationships with the equity of the food delivery brand which will subsequently generate a positive behavior toward the platform’s brand. Tsai et al. (2022) revealed that performance expectancy, effort expectancy, and security are the factors affecting the food delivery apps selection decision. Table 1 summarizes the articles addressing economic sustainability with the consideration of customer behaviors and preferences.

**Table 1:** Summary of the articles addressing economic sustainability with the consideration of consumer behaviors and preferences (*Remark: E* = *Empirical; A* = *Analytical)*

Article	Topic	Focused Economy	Research Approach	Research Methods	Theory/ Model Adopted	Significant Factors
Jha (2022)	Customer’s comments analysis to improve customer relationship	N/A	E	Optimization formulation; Case studies	N/A	N/A
Gunden et al. (2020)	Consumer behavior of using food delivery services	USA	E	Collected 605 consumer surveys; Using SEM	Extended Unified Theory of Acceptance and Use of Technology (UTAUT2)	Performance expectancy; Congruity with self-image; Habit; Mindfulness; Impulse purchase tendency
Francioni et al. (2022)	Consumer behavior of using food delivery services	Italy	E	Collected 360 consumer surveys; Using SEM	N/A	Perceived healthiness; Perceived hygiene; Quarantine procedures; Perceived ease of app use; Attitude
Wang and He (2021)	Consumer behavior of using food delivery platform services	China	E	Case study; Conducted public data analysis	Negative binomial model; Regression	N/A
Yen (2022)	Consumer behavior of using food delivery platform services	USA	E	Collected 217 consumer surveys; Using PLS-SEM	Relationship Theory; Brand Equity	Perceived economic exchange; Perceived social exchange; Mutual interests
Kapoor and Vij (2018)	Consumer behavior of using food delivery apps	India	E	Collected 350 consumer surveys; Conducted 2 focus group interviews; Using SEM	Attribute-Conversion model	Visual; Navigational; Information; Collaboration designs
Kaur et al. (2021)	Consumer behavior of using food delivery apps	India	E	Conducted focus group discussion with 20 app users; Applied grounded theory; Collected 423 consumer surveys; Using SEM	Theory of Consumption Value	Visibility, Affordances, Value for money; Prestige social benefit
Yeo et al. (2021)	Consumer behavior of using food delivery apps	Malaysia	E	Collected 250 consumer surveys; Using SPSS; and PLS-SEM	N/A	Perceived usefulness; Social influence; Trust
Raza et al. (2022)	Consumer behavior of using food delivery apps	Pakistan	E	Collected 836 consumer surveys; Using SEM	Trust Transfer Theory	Trust disposition; Online review
Ahn and Kwon (2021)	Consumer behavior on food delivery brand	Taiwan	E	Collected 577 consumer surveys; Using SEM	Stimulus-Organism-Response (SOR) model	Perceived usefulness; Perceived enjoyment; Perceived price; Customer’s innovativeness; Customer’s experience
Tsai et al. (2022)	Consumer behavior in selecting the order delivery platform	Taiwan	E	Conducted consumer interviews; Applied DEMATEL; DANP; and modified VIKOR for analysis	Extended Unified Theory of Acceptance and Use of Technology (UTAUT2)	Performance expectancy; Effort expectancy; Social influence; Facilitating conditions; Hedonic motivation; Habits; Security

#### Operational strategies and performances

In addition to customer behaviors, the existing literature has also examined different operational problems (e.g., routing planning, operational modes and optimal strategies) of food delivery services. For example, Lang and Zhao (2021) proposed a cloud computing resource scheduling approach for forecasting the take-out order quantity. Besides, by studying the route planning problem of the food delivery services, Du et al. (2019) developed a spatial crowdsourcing-based system, namely, crowd delivery network (CrowDNet) with the consideration of delivery cost and time trade-off. The proposed system consists of a hitchhiking ride service served by taxis and a ranking module for the delivery route planning. Kohar and Jakhar (2021) proposed an augmented 2-index formulation to determine the optimal food delivery routing by considering the constraints of time windows of both the restaurants and customer locations, and the capacity constraints of the fleets of vehicles. In addition, Wang et al. (2021) proposed an Extreme Gradient Boosting-enhanced (XGBoost-enhanced) algorithm to generate the routing solution within a limited computational time and minimal total cost. Zheng et al. (2022a, 2022b) studied a stochastic online food delivery problem (SOFDP) by developing the route planning and order assignment solutions. The solutions are characterized by large search space, strong coupling, capturing the uncertain food preparation time, and fulfilling the speedy evaluation requirements. Interesting, Ulmer et al. (2021) proposed an anticipatory customer assignment (ACA) policy by addressing delivery delay avoidance and random restaurant ready times. The proposed policy is featured by having postponement on customer’s assignment, including time buffer to avoid delay delivery, and bundling operations.

Another well-examined problem is related to the operational strategies of food delivery services. Taylor (2018) investigated the effect of delay sensitivity and agent independence on the platform’s optimal service price and wage in both static and uncertain customer’s valuation or agent’s opportunity cost. The analytical results demonstrated that the uncertainties of customer valuation and agent cost affect the optimal service price differently when considering the customer delay sensitivity and agent independence. Agent independence reduces the service price if the agent opportunity cost uncertainty is high, or the customer valuation uncertainty is low. Besides, delay sensitivity increases the optimal service price if the customer valuation uncertainty is moderate. Dai and Liu (2020) analyzed the optimal workforce capacity and order allocations among three different O2O delivery modes, namely in-house delivery, full-time crowdsourced riders, and part-time crowdsourced riders. Du et al. (2021) examined four different delivery strategies in either restaurant-operated or third party-operated platforms, and then derived the pricing decisions of each strategy. They found that the advertising effect from the restaurant delivery and the consumer benefit from third-party platform’s promotion will affect the optimal decisions of the restaurant to select the delivery strategy. Zhu et al. (2022) investigated the impacts of cooperating with the social media platform from the food delivery platform’s perspective. Their analytical results demonstrated that such cooperation would benefit the food delivery platform, but the users may have to bear higher registration fees. In addition, the authors applied the Nash negotiation framework to design a profit-sharing scheme such that both the food delivery platform and social media platform can be better-off. Jia et al. (2022) determined whether or not a restaurant should form partnership with the third-party delivery platform and statistically analyzed the optimal number of riders during the coronavirus pandemic based on the public data. Lin et al. (2022) explored the food delivery system under regular and physical internet driven self-delivery, outsourcing-delivery and volunteer-delivery business models. They developed a multi-objective mixed-integer linear programming (MOMILP) which aim to minimize the total cost but maximize the customer service level. The experiment results illustrated that the adoption of physical internet can enhance the performance of all three business models, but it generates greatest value to the volunteer-delivery business model. He et al. (2019) designed an agent based O2O food ordering model to investigate how the customer’s and platform’s behaviors affect the location and food quality decisions of the restaurant. The numerical analysis revealed that the customer’s food quality preference and the platform’s routing planning have a positive effect on the restaurant’s food quality decision and location decision, respectively. In addition, when the online platform is cost-saving conscious, it will affect the waiting time of the customers and the location decision of the restaurants.

The operational decisions of the food delivery platforms will affect their profitability, and it is important to quantify it. For example, Seghezzi and Mangiaracina (2020) examined the financial performance of the last-miles deliveries in the on-demand food delivery industry. They constructed a model to evaluate the profitability of the food delivery platform. The sensitively analyses showed that there is a fixed delivery price threshold that can make the food delivery platform business profitable regardless of the daily demand. Besides, having a higher demand does not necessarily help improving the profit of the food delivery platform as it may increase the delivery cost when the number of destinations increases. Sun et al. (2022) and Bai and Tang (2022) individually built analytical models to explore the impacts of having price and lead-time competition in food delivery platform operations on profitability. Sun et al. (2022) found that single dimensional competition with either price or lead-time will harm the profitability of the food delivery platform. However, under the joint price and lead-time competition situation, the platform will be better off if the intensities of pricing and lead time competitions are different. Apart from competing on price and lead-time, Bai and Tang (2022) also considered the platforms can offer higher wages to attract more riders to join. They showed that only one platform can result in a “payoff dominant stable equilibrium” who will take all the benefit. Feldman et al. (2022) explored a congested service system of the restaurant and analytically analyzed the supply chain performance under the commonly seen simple revenue-sharing contract in a supply chain with one restaurant and one third-party delivery platform. They concluded that a simple revenue-sharing contract is inefficient to coordinate the supply chain and harms the profitability of the restaurant. However, a properly set generalized revenue-sharing contract which consists of shared revenue and fixed fee sharing can help achieve supply chain coordination. Table 2 summarizes the articles addressing economic sustainability with the consideration of different operational problems and performances.

**Table 2:** Summary of the articles addressing economic sustainability with the consideration of various operational strategies and performances (*Remark: E* = *Empirical; A* = *Analytical)*

Article	Topic	Research approach	Research methods	Theory/ model adopted
Lang and Zhao (2021)	Take-out order forecasting	E	Mathematical formulation; Conducted experiments	Improved artificial neural network (ANN) model
Du et al. (2019)	Route planning of the food delivery	E	Optimization formulation; Conducted experiments	Modern portfolio theory; Insertion check algorithm; Local block scheduling algorithm
Kohar and Jakhar (2021)	Route planning of the food delivery	E	Mathematical formulation; Conducted experiments	Mixed integer linear programming model; Branch-and-cut search
Wang et al. (2021)	Route planning of the food delivery	E	Mathematical formulation; Using machine learning and constructive heuristics	Classification model; XGBoost-enhanced algorithm
Zheng et al., (2022a, 2022b)	Route planning and order assignment	E	Mathematical formulation; Machine learning modelling; Conducted experiments	Iterated greedy algorithm
Ulmer et al. (2021)	Customer assignment policy	E	Mathematical formulation; Conducted experiments	Route-based Markov decision process (MDP)
Taylor (2018)	Operational decisions of food delivery system	A	Analytical modelling	Game-theory; Queueing theory
Dai and Liu (2020)	Operational strategy of food delivery system	E	Mathematical formulation; Conducted experiments	Integer linear programming; Branch and bound algorithm
Du et al. (2021)	Operational strategy of food delivery system	A	Analytical modelling	Game-theory; Stackelberg game
Zhu et al. (2022)	Operational strategy of food delivery system	A	Analytical modelling	Game-theory; Nash bargaining framework
Jia et al. (2022)	Operational strategy of food delivery system	E	Optimization formulation; Conducted experiments	Stochastic integer programming; Susceptible-infected-recovered (SIR) model; Autoregressive-moving-average (ARMA) regression model
Lin et al. (2022)	Operational strategy of food delivery system	E	Mathematical formulation; Conducted experiments	Multi-objective mixed-integer linear programming (MOMILP) models
He et al. (2019)	Operational decisions of the restaurants	E	Estimation-and-optimization formulation; Conducted experiments	Agent-based model; Regression model
Seghezzi and Mangiaracina (2020)	Financial performance of the last-miles deliveries in the food delivery services	E	Conducted 3 interviews with managers working in food delivery platforms; Developed algorithms; Conducted experiments	N/A
Sun et al. (2022)	Financial performance of the food delivery platform with joint price and lead-time competition	A	Analytical modelling	Game-theory; Queueing theory
Bai and Tang (2022)	Financial performance of the food delivery platform with price and lead-time competition, and wage offering	A	Analytical modelling	Game-theory; Queueing theory
Feldman et al. (2022)	Supply chain performance of using revenue-sharing contract in the food delivery business model	A	Analytical modelling	Game-theory

### Social sustainability

Social sustainability relates to the benefits and welfares of the community (Hess et al., 2022). In the food delivery operations, it involves customers to engage in the food ordering process and requires riders to provide prompt delivery services. Therefore, the welfares of both the customers and riders are vital in the social sustainability.

#### Labor control

There are numerous articles studying various issues related to the welfares of workers and riders such as autonomy, and fairness. For instance, Galière (2020), Veen et al. (2020), Gregory and Sadowski (2021), Franke and Pulignano (2021), Heiland (2021), and Shanahan and Smith (2021) examined the labor control mechanism in the food delivery industry. To be specific, Galière (2020) revealed that food delivery platform executes the governmentality dispositive subjectification techniques to create a hyper-meritocratic ideal of justice such that the riders may give consent to the algorithmic management. Veen et al. (2020) showed that riders are monitored via the apps, provided with limited choices (as information is asymmetric), and work in an unclear performance management system. This finding is similar to Franke and Pulignano (2021)’s study in which the food delivery platform withholds the information and executes rules and regulations to increase its power for controlling the stakeholders and creating its value. Gregory and Sadowski (2021) found that riders have to do self-investment to “fit for work” and trade the autonomy for algorithmic dispatch. Both algorithmic management and temporal control are the regimes executed by the food delivery platform on the riders (Heiland, 2021). Shanahan and Smith (2021) also highlighted that the food delivery platform uses the unilateral modification of exchange terms, communication and technology designs, and neoliberalism and tribalism to force the riders to accept the job obligations. Table 3 summarizes the articles addressing social sustainability with the consideration of labor control.

**Table 3:** Summary of the articles addressing social sustainability with the consideration of labor control (*Remark: E* = *Empirical; A* = *Analytical)*

Articles	Topic	Focused economy	Research approach	Research methods	Theory/ model adopted	Significant factors
Galière (2020)	Labor control (Algorithmic management)	France	E	A case study consisted of interviews with 21 riders and observations; Applied grounded theory	Foucauldian Framework	N/A
Veen et al. (2020)	Labor control (Algorithmic management)	Australia	E	A case study consisted of interviews and one focus group interview with total 58 riders	Labor Process Theory	N/A
Gregory and Sadowski (2021)	Labor control (Algorithmic management)	Scotland	E	Conducted interviews with 25 riders	N/A	N/A
Franke and Pulignano (2021)	Labor control	Belgium	E	Conducted interviews with 37 riders; Using Abductive approach	Marxian Theory	N/A
Heiland (2021)	Labor control	Germany	E	Conducted interviews with 35 workers; Performed ethnography; Conducted survey	Labor Process Theory	N/A
Shanahan and Smith (2021)	Fairness and labor control	UK and France	E	A case study consisted of interviews with 12 riders and performed observations	Psychological Contract Theory; Lukes’ Theory of Power	N/A

#### Work conditions

To better understand the job quality, work conditions and work safety of riders, Goods et al. (2019) suggested that workers have to comprehend individual circumstances, job opportunity, and socio-political. Furunes and Mkono (2019) found that riders have both positive and negative work experiences. The negative work experiences of the riders mainly come from their salary and the challenges from communicating with all the restaurants, employer, and customers. Le Breton and Galiere (2022) observed that riders rely on the online peer discussion groups for knowledge sharing, symphonizing and shaping in social learning process. In practice, Sun et al. (2021) identified that riders are required to perform duty at fixed schedule and such de-flexibilization is caused by the labor management tactics, technological-driven operations, and cultural normalization of platform dependency. Piasna and Drahokoupil (2021) reported that riders are willing to work in a regular schedule basis and prefer to be self-employed. Besides, riders will base on the autonomy level, job market vulnerability and economic attachment of the food delivery platform to select the employment status and work schedule. Behl et al. (2022) concluded that the major obstacles of being a rider include high competition as well as long login hours and late-night delivery services provision, followed by poor remuneration and unfavorable conditions for getting incentives. Moreover, Puram et al. (2021) found that rider faces different work pressures and difficulties especially during the COVID-19 pandemic, including operational, customer-related, organizational, and technological issues. By comparing the working conditions between regional and international platforms, Muszynski et al. (2022) illustrated that the regional food delivery platforms evade price competition with the international ones, but they provide better working conditions (e.g., higher safety precautions) than that of the international ones due to the country’s specific work regulations. In addition to the work conditions, work safety of the riders should be considered. Zheng et al. (2022a, 2022b) found that the amount of delivery orders and the rider’s occupational injuries have an inverted U-sharped form, and the work pressure plays as a mediator between such relationship. To improve the welfares of the riders (such as reducing the occupational injuries or traffic accidents), government can play a role in the food delivery system. For instance, Fan et al. (2022) analytically showed that spot check and information publicity policies are effective measures to reduce traffic violation of the riders. However, the spot check policy would induce more fines on overdue delivery of the food delivery platform. Table 4 summarizes the articles addressing social sustainability with the consideration of work conditions.

**Table 4:** Summary of the articles addressing social sustainability with the consideration of work conditions (*Remark: E* = *Empirical; A* = *Analytical)*

Articles	Topic	Focused economy	Research approach	Research methods	Theory/ model adopted	Significant factors
Goods et al. (2019)	Perceived job quality of the riders	Australia	E	A case study consisted of interviews with 58 riders	Job Quality Framework	N/A
Furunes and Mkono (2019)	Experiences of riders on food delivery	N/A	E	Collected 330 customers online reviews from food delivery platforms; Collected 24 youtube video; Conducted content analysis and thematic analysis	Role and Script Theory	N/A
Le Breton and Galiere (2022)	Learning mechanisms of the riders in the food delivery service industry	France	E	Ethnographic study consisted of interviews with 40 workers, observations and lexicometric analysis	Social Learning Theory	N/A
Sun et al. (2021)	Perceived work flexibility of riders	China	E	Conducted interviews with 72 riders and platform workers; Collected 2111 rider surveys; Using ethnographic fieldwork; Applied grounded theory	N/A	N/A
Piasna and Drahokoupil (2021)	Work preference of the riders	Belgium	E	Collected administrative records from 3279 riders; Collected 554 rider surveys	N/A	N/A
Behl et al. (2022)	Work conditions of the food delivery platform	India	E	Using interpretive structural modelling to extract variables; Conducted MICMAC analysis	N/A	Operating expenses; Social protection & retirement benefits; Payment structure; Incentives; Login hours & late-night deliveries; High competition
Puram et al. (2021)	Difficulty of the last-mile food delivery	India	E	Conducted interviews with 38 riders; Applied grounded theory	N/A	Operational issues (cost, quality of work life, service design); Customer-related issues (co-creation; Communication; Delivery experience; organizational issues (ethics, policy); Technological issues (device, navigation)
Muszynski et al. (2022)	Work conditions of the food delivery platform	Poland and Italy	E	Case studies	N/A	Working conditions (contractual terms, salary, working duration, work intensity and work safety)
Zheng et al., (2022a, 2022b)	Work safety of the riders	China	E	Collected 9133 rider surveys	Self-Control Theory; Binary probit model	Order delivery amount
Fan et al. (2022)	Policy for reducing traffic violation	N/A	A	Analytical modelling	Game-theory; Stackelberg game	N/A

#### Worker’s commitment

Finally, Lin et al. (2020) and Lee et al. (2022) evaluated different factors affecting the work commitment and work performance of the riders. Specifically, Lin et al. (2020) statistically presented that work centrality, entitlement norms, obligation norms, and intrinsic orientation are positively associated with work engagement, which will subsequently generate a positive impact on the work commitment of the riders in the food delivery industry. Recently, Lee et al. (2022) showed that low levels of work commitment of the crowdsourced riders and perceived risks of the crowdsourcing technical systems are positively associated, and they eventually lead to a lower level of work performance and intention to work continuously in the food delivery system. However, the food delivery platform can provide support, develop a trust relationship and facilitate information sharing to mitigate the negative impacts of the perceived risks of crowdsourcing technical systems. Table 5 summarizes the articles addressing social sustainability with the consideration of worker’s commitment.

**Table 5:** Summary of the articles addressing social sustainability with the consideration of worker’s commitment (*Remark: E* = *Empirical; A* = *Analytical)*

Articles	Topic	Focused Economy	Research Approach	Research Methods	Theory/ Model Adopted	Significant Factors
Lin et al. (2020)	Work engagement of riders	China	E	Collected 50 interviews with riders; Collected 800 rider surveys	N/A	Work centrality; Entitlement norms; Obligation norms; Intrinsic values
Lee et al. (2022)	Rider's intention to work continuously in the food delivery industry	South Korea	E	Collected 267 rider surveys; Using Structural Equation model	Sociotechnical Systems Theory	Job commitment; Perceived difficulty of task requirement; Perceived technology complexity; Crowdsourced Job performance; Intention to work continuously

### Environmental sustainability

Environmental sustainability has a focus on mitigating the negative impacts to the environment, such as reducing carbon emission, recycling wastes, and using renewable energy. Despite it is an important area to be studied, we observed that there is limited article addressing the environmental sustainability in food delivery operations. To be specific, Liu et al. (2020) examined the packaging waste problem that is generated from the food delivery operations and evaluated its environmental impacts. By applying the big data mining, their findings revealed that plastic bags contribute most to the food packaging waste in the food delivery services and the use of paper boxes harm the environment most in terms of the quantity of carbon dioxide emission during the production process. Besides, the distribution of the pollution and distribution of food delivery service providers are positively associated. Table 6 summarizes the reviewed articles addressing environmental sustainability.

**Table 6:** Summary of the reviewed articles addressing environmental sustainability (*Remark: E* = *Empirical)*

Articles	Topic	Focused Economy	Research Approach	Research Methods	Theory/ Model Adopted	Significant Factors
Liu et al. (2020)	Food packaging waste and its environmental impact	China	E	Using Python based web-crawling and survey for big data mining; Applied life cycle impact assessment	N/A	N/A

### Muti-dimensional sustainability

In real-world practices, business firms would address more than one sustainability dimension depending on the levels of stakeholder involvement and stakeholder expectations (Fischer et al., 2020), as well as their goals. In the following, we review the articles addressing multi-dimensional sustainability in the food delivery operations and summarize them in Table 7.

**Table 7:** Summary of the articles addressing multi-dimensional sustainability (*Remark: E* = *Empirical; A* = *Analytical)*

Articles	Topic	Focused Economy	Sustainability Aspects Addressed	Research Approach	Research Methods	Theory/ Model Adopted	Significant Factors
Wang (2022)	Operational strategy on supervision of the O2O food delivery and the benefits of stakeholders	N/A	Economic & social	A	Analytical modelling	Tripartite evolutionary game; Simulation analysis	N/A
Chen et al. (2022)	Supply chain contracting in the food delivery business model with the consideration of the surplus of stakeholders	N/A	Economic & social	A	Analytical modelling	Game-theory; Stackelberg game; Queueing model	N/A
Niu et al. (2021)	Optimal operational strategy on delivery and impacts on the environmental performance	N/A	Economic & environmental	A	Analytical modelling	Game-theory; Bertrand competition model	N/A
Chen and Lee (2022)	Consumer's green behavior in using eco-friendly OFD platform	China	Economic & environmental	E	Collected 445 customer surveys	Legitimacy theory; Self-identity theory	Green brand legitimacy; Biosphere value Orientation; trust in green brands, Warm glow, Self-expressive benefits; Nature experiences, Utilitarian environmental benefits
Moncef and Dupuy (2021)	Paradoxical tensions on environment and social dimension in last-mile deliveries	N/A	Social & environmental	E	Case studies consisted of 27 interviews	N/A	N/A
Sinha and Pandit (2021)	Environmental and social impacts of hyper-local food delivery services	India	Environment & Social	E	Agent-based simulation	N/A	N/A
Seghezzi et al. (2021)	A review on the inter-relationship and values of food delivery	N/A	Economic, social & environment	E	Literature review based on 59 papers from 2016 to 2020; and conducted 4 interviews with practitioners	N/A	N/A

#### Economic and social sustainability: Operational strategies and social welfare

Wang (2022) and Chen et al. (2022) studied both economic and social sustainability performances in the food delivery operations. To be specific, Wang (2022) examined the best operational strategy of the food delivery platform by considering the bounded rationality and the expected benefits of the supply chain members including restaurants, food delivery platform and customers. The author showed that food delivery platform should penalize the restaurants heavily if they violate the regulations. Besides, the food delivery platform should also use the supervision strategy if the total supervision cost is lower than that of the negative social evaluations under the non-supervision strategy. Chen et al. (2022) analytically analyzed the value of collaborating with the online food delivery platform from the restaurant’s perspective under the revenue-sharing contract and investigated the way to achieve supply chain coordination. They showed that collaborating with the online food delivery platform is not necessarily beneficial to the restaurant in increasing the demand. Both the platform and the restaurant will be better off if a revenue-sharing contract with a price ceiling or a two-way revenue-sharing contract are adopted. When the supply chain is not coordinated, the platform can increase its profit and social welfare through better controlling its number of riders.

#### Economic and environmental sustainability: operational strategies and environmental pollution

Regarding the economic and environmental considerations in the food delivery operations, Niu et al. (2021) first evaluated the optimal pricing mechanism under platform’s delivery strategy and restaurant’s self-delivery strategy, and then examined their impacts on restaurant’s financial performance and supply chain’s environmental sustainability. The authors found that restaurant prefers adopting platform’s delivery strategy when the market demand is low. Besides, the platform’s delivery strategy is more environmentally sustainable than that of the restaurant’s self-delivery strategy when the market demand is high. In addition, Chen and Lee (2022) identified how the environmental performance of a food delivery platform shapes the customer behavior on it. They statistically found that green brand legitimacy and perceived biosphere value orientation have a significant positive impact on the customer’s trust in the food delivery platform which will eventually lead to a positive consumer behavior in using the environmentally friendly platform.

#### Social and environmental sustainability: social welfare and environmental pollution

Last but not least, Moncef and Dupuy (2021) and Sinha and Pandit (2021) considered both social and environmental sustainability performances in the food delivery operations. Moncef and Dupuy (2021) investigated the paradoxical tensions faced by different sharing economy in logistics management. They concluded that the food delivery platforms should put more resources in lowering the carbon dioxide emission in the delivery process. Besides, they should also improve the work conditions, and provide better policies and support to the riders. Interestingly, Sinha and Pandit (2021) quantified the amount of environmental pollution generated from the food delivery and the workload of the riders. By simulating 2100 customer food orders, it was found that about 163 gm carbon dioxide is emitted for each order delivery, and 15 orders are handled by each rider with idle time of about 59.2%.

#### Economic, environmental and social sustainability: inter-relationship and values of food delivery

There is limited literature addressing all the economic, environmental and social sustainability challenges together in food delivery operations. Seghezzi et al. (2021) conducted a literature review on the on-demand food delivery to identify the roles of each player and explore the value-adding activities in the food delivery operations. Moreover, the authors conducted interviews with practitioners to uncover the underexplored research areas. Overall, they showed that operations activities (such as food preparation) and restaurant’s benefits require an in-depth analysis as the current discussion on these areas in the literature is insufficient.

## Discussions and future research agenda

**Fig. 5:**
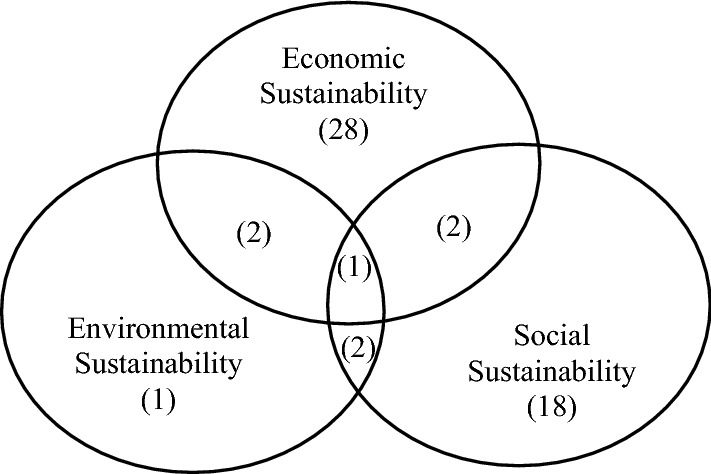
Distribution of the reviewed literature in the TBL framework. (Remarks: The number indicated in the bracket represents the number of articles)

After reviewing the relevant literature, we have identified three research areas that are underexplored in the field of third-party food delivery operations. See Fig. 5 and Table 8 for the details. First, there is inadequate investigation on the restaurant’s preferences and decisions in the third-party food delivery system. Referring to the economic sustainability literature, Jia et al. (2022) is the only study which examined the restaurant’s decision on forming partnership with the third-party food delivery platform. The number of partner restaurants in a food delivery platform may affect the customer’s incentive to use delivery services, and hence the profitability of the food delivery platform. It is crucial to understand the restaurants’ preferences, concerns and decisions as it will affect the success of a third-party food delivery platform. Second, the understanding on environmental performance in the third-party food delivery operations literature is insufficient. It is found that the impacts of food packaging and waste in the food delivery service was addressed only in Liu et al. (2020). In practice, the food delivery platforms have developed various environmentally sustainable programs to reduce carbon emission and pollution. However, the studies related to food delivery platform operational green practices, and the customer’s attitude and behavior towards such green practices have not been well explored. Finally, the examination on the multi-dimensional sustainability in the third-party food delivery operations is very limited. Each dimension in TBL is inter-related. For example, the delivery strategy of the food delivery operations will affect the environmental sustainability performance (Niu et al., 2021) while a proper design of a supply chain contract between the restaurant and food delivery platform will affect the food delivery platform’s profitability and social welfare (Chen et al., 2022). It is essential to consider the benefits of supply chain members, impacts on the environment, as well as the food delivery platform’s financial performance for its sustainable growth and development.

**Table 8:** Distribution of the research topic

Sustainability aspect(s)	Topic	Restaurants	Third-party food delivery platform	Riders	End customers	Supply chain
Economic	Consumer behaviors and preferences				11	
	Operational strategies and performances	1	11			5
Social	Labor control			6		
	Work conditions			10		
	Worker's commitment			2		
Environmental	Food packaging and waste		1			
Economic and social	Operational strategies and social welfare					2
Economic and environmental	Operational strategies and Environmental pollution					2
Social and environmental	Social welfare and environmental pollution					2
Economic, social and environmental	Inter-relationship and values of food delivery					1

In the following, we propose a future research agenda on five different areas, namely, applications of digital technologies, behaviors and decisions of the restaurants, risk management, TBL, and post-coronavirus pandemic. Table 9 summarizes the future research agenda proposed in this study.

**Table 9:** Future research agenda proposed in this study

Areas	Real-world practice and observation	Proposed topics
Applications of digital technologies	Driverless autonomous delivery	To examine the perceived value, experience, and preference of the end-customers of the driverless autonomous delivery To investigate the value of using the autonomous delivery by comparing with the traditional operations in terms of profitability, consumer utility and environmental performance
	Blockchain supported token payments and smart contracting	To identify the determinants of using the blockchain technology in the food delivery operations from both end-customers’ and restaurants’ perspectives and understand what kind of support that should be provided by the third-party food delivery platform To present the business models of blockchain-supported operations and analyze the impacts of using smart contracts to facilitate consumer reviews valuation and rewards programs in the food delivery operations
Behaviors and decisions of the restaurants	Some restaurants form partnership with one food delivery platform only	To examine the determinants in selecting the food delivery platform and evaluate the benefits of such selection To empirically explore the effects of forming business partnership with more than one food delivery platforms and the values and risks of such practices To analytically derive the optimal operational strategy of the restaurant, including the optimal number of business partnerships with the food delivery platforms and the optimal contract setting with the consideration of the competition between platforms
Risk management	Logistics risk	To develop new algorithms to address the issue of logistics risks in route planning To study possible alternative operational strategy that can enhance the capacity flexibility of the food delivery platform
	Food quality risk	To investigate the effect of food quality risk arose from the last-mile delivery on the restaurant’s brand equity To apply machine learning and data analytics to estimate the food delivery time and food temperature which can help the restaurants to develop measurements to maintain the food quality
	Profit risk	To compare different business partnership models in the real-world situation, and then analytically determine the best operational strategy in the food delivery operations
Triple bottom line (TBL)	Food delivery platform provides rebate on the commission fees in economic sustainability	To conduct risk sensitivity estimation of the business firm to enhance the operational efficiency of the on-demand platform To examine other sophisticated supply contracting (e.g., considering the rebate component) and determine the win–win situation of the food delivery operations in the economic sustainability
	Food delivery platform executes electric vehicles for food delivery in environmental sustainability	To understand the customers behaviors in using the “green” food delivery platform To construct analytical models to evaluate the performance of the green practices under different environmental schemes imposed by the government (e.g., carbon tax, cap-and-trade)
	More than a thousand cases of the delivery accidents	To investigate whether the government should provide subsidy or issue penalty scheme to the food delivery platforms to ensure the safety of the riders in last-mile delivery and explore its impacts in the supply chain performance
Post-coronavirus pandemic	“Ghost kitchen” business model	To study the future needs of the customers in the post-coronavirus pandemic era and evaluate whether there is a new business model for the food delivery platforms to survive after coronavirus pandemic To identify the drivers of using the food delivery platform, conduct a customer demand analysis and analyze the operational decisions in the post-coronavirus pandemic

### Applications of digital technologies

Information sharing is a crucial element in achieving sustainable operations (Zhang et al., 2018). With the emergence of disruptive technologies in Industry 4.0 such as blockchain (Luo & Choi, 2022), Internet-of-Thing (IoT), and robot (Sheu & Choi, 2022), it will affect the existing food delivery operations, enable an efficient information sharing among all stakeholders and facilitate novel business models (Akter et al., 2022). For example, Uber Eats has used robots to support driverless autonomous delivery and order tracking services to the end-customers in the US. It is believed that such services are time and cost efficient as the end-customers can get their meal in a quicker manner and save tip costs paid to the riders. From the perspective of Uber Eats, the autonomous delivery reduces the number of riders requirement, and is more eco-friendly than that of the traditional car delivery.^6^ However, it will also require labours to monitor the operations and a higher level of customer involvement in the operations as the robot is not able to enter an apartment.^7^ Since this is a new service, in the future, it is promising to examine the perceived value, experience and preference of the end-customers on the driverless autonomous delivery. Besides, it deserves our efforts to analytically investigate the values of using the autonomous delivery in the third-party food delivery operations by comparing with the traditional operations in terms of profitability, consumer utility and environmental performance.

Another disruptive technology that has been applied in the food delivery operations is the blockchain technology (Choi & Shi, 2022). Blockchain technology is regarded as a distributed digital ledger that improves information transparency, ensures food safety and hygiene, and supports faster and secure payment (Choi et al., 2022a, 2022b). In real-world example, Bistroo has adopted the blockchain technology in its food delivery ecosystem to facilitate token payments and smart contracts. As the token payments do not involve intermediates, Bistroo is able to lower the commission fee charged to the restaurant, which will result in a more competitive pricing setting. Besides, by using the blockchain technology, customers can acquire trusted and reliable information about the food quality and restaurant rating, and are beneficial from the customer rewards programs which are enabled by the blockchain-supported smart contracts.^8^ However, the success of applying the blockchain technology in the food delivery operations requires the support from both the end-customers and restaurants, as well as the investment from the food delivery platform. Thus, it is important to identify the determinants of using the blockchain technology in the food delivery operations from both the end-customers’ and restaurants’ perspectives and then understand what kind of support that should be provided by the food delivery platform. On the other hand, one may consider illustrating such business model, and analyzing the impacts of using smart contracts to enable the consumer reviews valuation and rewards programs.

### Behaviors and decisions of the restaurants

The use of third-party food delivery app is a new trend during the coronavirus pandemic, and the corresponding perceived benefits and risks should be identified (Gupta & Duggal, 2020). In this field of study, the majority of prior studies consider the perspective of the individual end-customers only and there are very few studies emphasizing the driving forces of forming a partnership with different third-party food delivery platforms from the perspective of restaurants (Sin et al., 2021). In the food delivery operations, restaurants are also the business customers of the third-party food delivery app and play an important role in the food delivery business. By forming a partnership with the third-party food delivery platform, it can increase the customer base of the restaurants. In reality, we have observed that some restaurants form partnership with one food delivery platform only. See Table 10 for details. Therefore, one possible future research topic is to explore the behaviors and decisions of the restaurants. For example, it is interesting to examine the determinants in selecting the food delivery platform and then evaluate the benefits of such selection.

**Table 10:** Examples of Hong Kong’s food delivery platform business customers (Adapted from https://deliveroo.hk/en/about-us/ and https://www.foodpanda.hk/)

	Foodpanda	Deliveroo
McDonald	✓	✓
MOS Burger	✗	✓
Pizza Hut	✓	✗
PHD	✓	✓
KFC	✓	✓
Jollibee	✓	✗
Tamjai	✗	✓
TamJai SamGor Mixian	✓	✗

We take Hong Kong as an example as the information is more accessible

Moreover, after forming a partnership with the food delivery platforms, restaurants not only need to manage the operations of their own restaurants but also the food delivery apps. Restaurants, such as Presidio Pizza Company and Proposition Chicken in San Francisco, intended to form partnership with more than one food delivery platforms to increase revenues. However, they withdrew the use of the third-party food delivery apps as each platform’s app has its own hardware and software. This caused employees to shuffle multiple orders from iPads, platform device, phone and in-store customer lines (Houck, 2017). Therefore, it is also crucial to explore the effects of forming business partnership with more than one food delivery platforms and the values and risks of such practices. In addition, the decisions about the optimal operational strategy of the restaurant, including the optimal number of business partnerships with the food delivery platforms and the optimal contract setting with the consideration of the competition between platforms, deserve an in-depth investigation as well.

### Risk management

Risk management is undoubtedly an important issue in the service industry (Choi et al., 2016), however, it is underexplored in food delivery operations. Niu et al. (2021) stated that food and logistics are connected to food delivery. Food cannot be delivered from the restaurants to the end-customers without the logistics support. There are different types of risk in food delivery operations. The first one is logistics risk, which consists of “loss of food” and “late delivery”. For the loss of food, it can be either an entire order is missing, or part of an order is missing. Common reasons for missing items include restaurant’s false, rider’s false (Butler 2022) or customers’ false claim (Mok, 2021; Helling, 2022). Helling (2022) presented that restaurants may often make mistakes on order items when they have to handle multiple orders at once and rider may deliver the order to a wrong address. Therefore, “loss of food” will decrease the reliability of a food delivery platform and lower the consumer’s utility. Besides, Mok (2021) mentioned that certain customers make a false claim on “missing” food to take advantage of fraud. For instance, Uber Eats provides refund to the customers if they report the “missing” food items. However, it does not investigate the root cause of having “missing” food items but simply deduct such cost directly from the restaurant payouts (Mok, 2021). This will increase the profit risk of the restaurants. On the other hand, “late delivery” is another logistics risk. Uber Eats in Melbourne always faces the late delivery problem (Dexter, 2021). This may be due to the fact that the order assignments are scheduled by the algorithms in which factors such as rider’s driving experience are not well considered. Besides, customer may deliberately provide an incorrect delivery address to avoid high delivery fee (Spence, 2022) which will increase the deliver time as the riders need more time to search for the right location. Therefore, new algorithms should be developed to address the logistics risks in the route planning. Besides, alternative operational strategy that can enhance the capacity flexibility of the food delivery platform should be explored and its effects should be examined. Zhang et al. (2022) provide an analytical discussion on this area.

Furthermore, food quality risk is also an obstacle to the restaurants’ operations. It is difficult for the restaurants to control their food quality once their food is out of the restaurants (Marks, 2020). According to Currington (2022), 82% of customers complained about the restaurants instead of the riders or the food delivery platforms for the food quality problems. This illustrated that the uncontrollable food quality problem in the last-mile delivery may affect the restaurant’s reputation. In the future, it is interesting to investigate the impacts of food quality problem arose from the last-mile delivery on the restaurant’s brand equity. It is also important to apply machine learning and data analytics to estimate the food delivery time and food temperature which can help the restaurants to develop measurements to maintain the food quality.

Finally, profit risk should also be examined. Restaurants, including both giant chain restaurants or small restaurants such as teahouses and hawker stalls, may prefer to form partnership with food delivery platform who offers a lower commission fee. Deepak Kaul, an owner of an Indian restaurant in Portland, indicated that he has to pay about 30% commission fee to the food delivery platform which will result in a large variation in the restaurant’s profitability. Therefore, many restaurants encourage customers to order food delivery by calling the restaurants directly (Shah, 2020). It is promising to compare different business partnership models in the real-world situation, and then analytically determine the best operational strategy in the food delivery operations. To study risk management and quantify risk in the supply chain context, Choi et al. (2018) and Li et al. (2013) provide good discussion on building the related analytical models.

### Triple bottom line (TBL)

Economic sustainability is a crucial dimension in triple bottom line (TBL) framework as the business firms have to be profitable for survival in the competitive marketplace. In the existing literature, two articles analytically examined the supply chain performance under the revenue-sharing contract and proposed the mechanism to achieve supply chain coordination (Chen et al., 2022; Feldman et al., 2022). In real-world observation, we have found that third-party food delivery platform offers new subsidy scheme to restaurants to lessen their financial burden on the commission fee. For example, the platform, SkipTheDishes, provided 15% of rebate on the total commission fees paid by the restaurants during the coronavirus pandemic to increase the customer demand as the food price will be set lower (McLean, 2020). On the other hand, during the coronavirus pandemic, the business environment is highly dynamic which will significantly affect the profitability and performance of the supply chain. It is recommended to first conduct the risk sensitivity estimation of the business firm to enhance the operational efficiency of the on-demand platform (Choi & Shi, 2022). Apart from using revenue-sharing contract, other sophisticated supply contracting (e.g., a supply chain contract with rebate component) can be explored to determine the win–win situation of the food delivery operations. Chiu et al. (2011) provide a good reference in this area.

Regarding the environmental sustainability, it is found that limited literature addressed this dimension when studying the food delivery businesses. As the operations of the online food delivery platforms are driven and supported by the technology and big data, their environmental performance (e.g., carbon dioxide emission) can be evaluated based on new methods (Song et al., 2017; Yao et al., 2020). Besides, it is reported that the motorcycles last-mile delivery contributes to noise and air pollution if they are not using eco-friendly electric ones (García, 2022). In reality, Swiggy has planned to execute electric vehicles for food delivery^9^ to tackle this problem. Moreover, the well-established food delivery platform Foodpanda has implemented different measures to be environmentally sustainable including launching “reusable packaging” and “plastic containers recycling” programs.^10^ However, the food delivery platform needs to take initiative to invest in environmental sustainability measurement. In the future, it is suggested to conduct an in-depth investigation to understand the customers behaviors in using the “green” food delivery platform which will provide a good reference on the future development (such as the use of electric vehicles for food delivery, launching environmentally sustainable programs). Based on the findings, it can help to construct analytical models to evaluate the performance of the green practices under different environmental schemes imposed by the government (e.g., carbon tax, and cap-and-trade policy) (Homayouni et al., 2021; Sheu & Chen, 2012). The food delivery platform should strike a balance between the economic sustainability and environmental sustainability, where existing literature addressing multi-dimensions of sustainability is very few and deserve our further exploration.

Finally, in terms of the social sustainability, it is found that customers may experience service inconsistency between the restaurant and the food delivery platform (Furunes & Mkono, 2019) which will affect the consumer utility. In this case, both the restaurant and food delivery platform should seek for a mechanism to improve the situation and evaluate the benefits. Besides, there were more than a thousand cases of the delivery accident reported over the past years.^11^ In the future, one potential area for investigation is whether the government should provide subsidy or issue penalty scheme to the food delivery platforms to ensure the safety of the riders when providing the last-mile delivery, and explore its impacts on the supply chain performance.

### Post-coronavirus pandemic

Previous studies have discussed some findings on food delivery services and operations during the coronavirus (COVID-19) pandemic situation. However, to be successful in an industry, a long-term sustainable growth is much more important than focusing on the short-term development during the COVID-19 pandemic only (Li et al., 2020; Ivanov, 2022). It is observed that customers have changed their eating habit from dining out to dining at home since the time when governments have imposed social distancing restrictions during the pandemic. Therefore, there is a boom in the “ghost kitchen” business model that the restaurants prepare their meals from a “virtual” facility operated by the third-party food delivery companies (rather than in their own physical kitchens) and offer the delivery-only service to the customers. For example, Uber Eats has run more than 1500 ghost kitchens in the US and Canada, where customers can place an order from the restaurants’ physical kitchens or ghost kitchen of Uber Eats (Sherred, 2019). Under this virtual business model, the restaurants can save both the fixed cost (e.g., rental) and variable cost (e.g., waitress’s salary) in their operations. It is estimated that the market size of “ghost kitchen” will hit the level of level of USD139.37 billion by 2028 (AFP, 2021). However, if all the social distancing restrictions are cancelled after the coronavirus pandemic, customer’s preference may be different, and they are more likely to dine out of their home again. Therefore, the customer preference of ordering from the third-party food delivery platform as well as from the ghost kitchen during and after the coronavirus pandemic should be studied in the future (Li et al., 2018). For example, it is important to identify the key drivers of ordering from third-party food delivery platforms, conduct a customer demand analysis and analyze the operational decisions (e.g., optimal pricing decisions and optimal number of riders in the ghost kitchen model) in the post-coronavirus pandemic era.

## Concluding remarks

To conclude, third-party food delivery is in a rapid evolution especially during the coronavirus pandemic. To maintain a sustainable development of the food delivery businesses, it is important to satisfy both the customers’ short-term benefits and all stakeholders’ long-term values. This study first conducts a systematic literature review to identify how to achieve a sustainable operation for third-party food delivery. Then, it highlights the recent advances in this important area with the discussion of real-world practices.

In particular, in this study, we first examine the relevant literature and apply the triple bottom line (TBL) framework to classify prior studies into economic sustainability, environmental sustainability, social sustainability, and multi-dimensional sustainability. We then identify three major research gaps, including inadequate investigation on the restaurant’s preferences and decisions, insufficient understanding on the environmental performance, and limited examination on the multi-dimensional sustainability in third-party food delivery operations. Finally, based on the reviewed literature and observed industrial practices, we propose five future research areas that deserve an in-depth further investigation. These areas include the applications of digital technologies, behaviors and decisions of the restaurants, risk management, TBL, and post-coronavirus pandemic.

Overall, to achieve a sustainable operation for third-party food delivery, the food delivery businesses should commit to the economic, social and environmental responsibilities. Specifically, the food delivery platform should understand the customer’s behaviors and preferences in using the food delivery service. Besides, it has to carefully decide the optimal operational strategies (including the routing, pricing, and delivery mode), analyze the effect of market competition, and study the operational impacts of food delivery on the environment. Moreover, it needs to consider the worker’s (i.e., riders) benefits, engagement, and safety issue as well as cooperation scheme with the restaurants such that the food delivery businesses can sustain a long-term success. Last but not least, government’s policies will affect the operations of the food delivery service and the economic sustainability of entire food delivery system. For instance, the government of New South Wales has imposed laws to improve the safety for riders who should be provided with training and personal protective equipment (PPE).^12^ In China, government requires food delivery platforms to lower the commission fee charged to the restaurants (Qu, 2022). Therefore, the food delivery businesses should keep reviewing the government policies when making operational decisions.

We do admit some limitations of this study. First, the literature review is systematic and follows a well-defined logic. However, as we all know, some related papers may still be missing. Second, for the related practices, we have included many commonly seen and well-established operations as a support to our arguments. However, there are always exceptions and the practices of food delivery platforms in different cities/countries may differ. So, we do not claim that our discussions are universally true. Readers should understand these limitations when they interpret our results and proposals.

## Footnotes

1 https://www.woking.gov.uk/environmental-services/food-hygiene/coronavirus-takeaway-food-and-deliveries-guidance (accessed 8 August 2022).

2 https://yalantis.com/blog/three-types-of-on-demand-delivery-platforms-pros-and-cons/ (accessed 8 August 2022).

3 https://www.statista.com/outlook/dmo/eservices/online-food-delivery/platform-to-consumer-delivery/worldwide#:~:text=Revenue%20in%20the%20Platform%2Dto,US%24302.40bn%20by%202026 (accessed 8 August 2022).

4 https://www.epa.gov/sites/production/files/2015-08/documents/reducing_wasted_food_pkg_tool.pdf (accessed 2 December 2022).

5 The experts recommended 13 articles related to the food delivery operations. To ensure consistency, the author (who filtered the papers collected from Web of Science) read the abstract and introduction section of each article to determine their relevancy for review. All the recommended articles are relevant, and they are not duplicated in the initial searching.

6 https://www.makeuseof.com/how-uber-eats-new-autonomous-deliveries-will-work/ (accessed 8 August 2022).

7 https://www.inc.com/ben-sherry/ai-delivery-robots-autonomous-vehicles.html (accessed 8 August 2022).

8 https://bistroo.io/whitepaper (accessed 8 August 2022).

9 https://www.zeebiz.com/india/news-food-deliveries-on-electric-vehicles-how-swiggy-is-using-evs-to-deliver-15-million-orders-every-month-96963 (accessed 8 August 2022).

10 https://www.foodpanda.hk/zh/contents/sustainability-reduce-plastic-waste?r=1 (accessed 2 December 2022).

11 https://www.thestar.com.my/news/nation/2022/07/21/1242-food-delivery-rider-accidents-from-2018-to-may-2021 (accessed 8 August 2022).

12 https://www.nsw.gov.au/customer-service/media-releases/new-safety-laws-food-delivery-riders-need-to-know (accessed 18 January 2023).

## References

[CR1] AFP (2021). *“Ghost kitchens’ boom in Asia as pandemic sparks huge demand*. Hong Kong Free Press. Retrieved November 30, 2022, https://hongkongfp.com/2021/09/19/ghost-kitchens-boom-in-asia-as-pandemic-sparks-huge-demand/

[CR2] Ahn J, Kwon J (2021). Examining the relative influence of multidimensional customer service relationships in the food delivery application context. International Journal of Contemporary Hospitality Management.

[CR3] Ahuja, K., Chandra, V., Lord, V., & Peens, C. (2021). *Ordering in: The rapid evolution of food delivery*. McKinsey & Company. Retrieved August 30, 2022, https://www.mckinsey.com/industries/technology-media-and-telecommunications/our-insights/ordering-in-the-rapid-evolution-of-food-delivery.

[CR4] Akter S, Michael K, Uddin MR, McCarthy G, Rahman M (2022). Transforming business using digital innovations: The application of AI, blockchain, cloud and data analytics. Annals of Operations Research.

[CR5] Bai J, Tang CS (2022). Can two competing on-demand service platforms be profitable?. International Journal of Production Economics.

[CR6] Barthel P, Ivanaj V (2007). Is sustainable development in multinational enterprises a marketing issue?. Multinational Business Review.

[CR7] Behl A, Rajagopal K, Sheorey P, Mahendra A (2022). Barriers to entry of gig workers in the gig platforms: Exploring the dark side of the gig economy. Aslib Journal of Information Management.

[CR8] Bozkaya E, Eriskin L, Karatas M (2022). Data analytics during pandemics: A transportation and location planning perspective. Annals of Operations Research.

[CR9] Butler, S. (2022). *Delivery driver drops food off at wrong house then tells customer to fetch it himself*. Indy100. Retrieved August 30, 2022, https://www.indy100.com/viral/delivery-driver-food-wrong-house.

[CR10] Chau, C. (2021). *Hong Kong police warn striking Foodpanda workers to disperse or face possible force*. Hong Kong Free Press. Retrieved August 30, 2022, https://hongkongfp.com/2021/11/16/hong-kong-police-warn-striking-foodpanda-workers-to-disperse-or-face-possible-force/.

[CR11] Chen M, Hu M, Wang J (2022). Food delivery service and restaurant: Friend or foe?. Management Science, Published Online,.

[CR12] Chen X, Lee TJ (2022). Potential effects of green brand legitimacy and the biospheric value of eco-friendly behavior on online food delivery: A mediation approach. International Journal of Contemporary Hospitality Management.

[CR13] Chiu CH, Choi TM, Tang CS (2011). Price, rebate, and returns supply contracts for coordinating supply chains with price-dependent demands. Production and Operations Management.

[CR14] Choi TM (2022). Achieving economic sustainability: Operations research for risk analysis and optimization problems in the blockchain era. Annals of Operations Research.

[CR15] Choi TM (2022). Financing product development projects in the blockchain era: Initial coin offerings versus traditional bank loans. IEEE Transactions on Engineering Management.

[CR16] Choi TM, Kumar S, Yue X, Chan HL (2022). Disruptive technologies and operations management in the Industry 4.0 era and beyond. Production and Operations Management.

[CR17] Choi TM, Shi X (2022). On-demand-ride-hailing-service platforms with hired drivers during coronavirus (COVID-19) outbreak: Can blockchain help?. IEEE Transactions on Engineering Management.

[CR18] Choi TM, Wallace SW, Wang Y (2016). Risk management and coordination in service supply chains: Information, logistics and outsourcing. Journal of the Operational Research Society.

[CR19] Choi TM, Zhang J, Cheng TCE (2018). Quick response in supply chains with stochastically risk sensitive retailers. Decision Sciences.

[CR20] Chokshi, M. (2020). *Online food delivery apps: Why are they so much in demand?* CustomerThink. Retrieved August 1, 2022, https://customerthink.com/online-food-delivery-apps-why-are-they-so-much-in-demand/.

[CR21] Cini L, Goldmann B (2021). The worker capabilities approach: Insights from worker mobilizations in Italian logistics and food delivery. Work, Employment and Society.

[CR22] Crane A, Desmond J (2002). Societal marketing and morality. European Journal of Marketing.

[CR23] Currington, E. (2022). *Why 3rd-party delivery platforms are problematic for restaurants*. The Digital Restaurant. Retrieved August 16, 2022, https://thedigitalrestaurant.com/food-delivery-service-apps-problem-for-restaurants/.

[CR24] Dai H, Liu P (2020). Workforce planning for O2O delivery systems with crowdsourced drivers. Annals of Operations Research.

[CR25] Dexter, R. (2021). *Why is your Uber Eats order taking so long to arrive?* The Age. Retrieved August 16, 2022, https://www.theage.com.au/national/victoria/why-is-your-uber-eats-order-taking-so-long-to-arrive-20211111-p59824.html.

[CR26] Du J, Guo B, Liu Y, Wang L, Han Q, Chen C, Yu Z (2019). CrowDNet: Enabling a crowdsourced object delivery network based on modern portfolio theory. IEEE Internet of Things Journal.

[CR27] Du Z, Fan ZP, Gao GX (2021). Choice of O2O food delivery mode: Self-built platform or third-party platform? Self-delivery or third-party delivery?. IEEE Transactions on Engineering Management.

[CR28] Fan B, Lv L, Han G (2022). Online platform’s corporate social responsibility for mitigating traffic risk: Dynamic games and governmental regulations in O2O food delivery industry. Computers & Industrial Engineering.

[CR29] Feldman P, Frazelle AE, Swinney R (2022). Managing relationships between restaurants and food delivery platforms: Conflict, contracts, and coordination. Management Science.

[CR30] Fischer D, Brettel M, Mauer R (2020). The three dimensions of sustainability: A delicate balancing act for entrepreneurs made more complex by stakeholder expectations. Journal of Business Ethics.

[CR31] Francioni B, Curina I, Hegner SM, Cioppi M (2022). Predictors of continuance intention of online food delivery services: Gender as moderator. International Journal of Retail & Distribution Management.

[CR32] Franke M, Pulignano V (2021). Connecting at the edge: Cycles of commodification and labour control within food delivery platform work in Belgium. New Technology, Work and Employment.

[CR33] Furunes T, Mkono M (2019). Service-delivery success and failure under the sharing economy. International Journal of Contemporary Hospitality Management.

[CR34] Galière S (2020). When food-delivery platform workers consent to algorithmic management: A Foucauldian perspective. New Technology, Work and Employment.

[CR35] García, S. (2022). *The negative effects of food delivery: From pollution to malnutrition*. El Pais. Retrieved August 16, 2022, https://english.elpais.com/society/2022-06-29/the-negative-effects-of-food-delivery-from-pollution-from-malnutrition.html.

[CR36] Goods C, Veen A, Barratt T (2019). “Is your gig any good?” Analyzing job quality in the Australian platform-based food-delivery sector. Journal of Industrial Relations.

[CR37] Gregory K, Sadowski J (2021). Biopolitical platforms: The perverse virtues of digital labour. Journal of Cultural Economy.

[CR38] Gunden N, Morosan C, DeFranco A (2020). Consumers’ intentions to use online food delivery systems in the USA. International Journal of Contemporary Hospitality Management.

[CR39] Gupta V, Duggal S (2020). How the consumer’s attitude and behavioral intentions are influenced: A case of online food delivery applications in India. International Journal of Culture, Tourism and Hospitality Research.

[CR40] Gupta V, Sajnani M (2019). Risk and benefit perceptions related to wine consumption and how it influences consumers’ attitude and behavioral intentions in India. British Food Journal.

[CR41] He Z, Han G, Cheng TCE, Fan B, Dong J (2019). Evolutionary food quality and location strategies for restaurants in competitive online-to-offline food ordering and delivery markets: An agent-based approach. International Journal of Production Economics.

[CR42] Heiland H (2021). Neither timeless, nor placeless: Control of food delivery gig work via place-based working time regimes. Human Relations.

[CR43] Helling, B. (2022). *DoorDash missing item? Here’s what to do*. Ridester.com. Retrieved August 16, 2022, https://www.ridester.com/doordash-missing-item/.

[CR44] Hess D, Rogovsky N, Dunfee TW (2002). The next wave of corporate community involvement: Corporate social initiatives. California Management Review.

[CR113] Høgevold, N. M., Svensson, G., Wagner, B., J. Petzer, D., Klopper, H., Carlos Sosa Varela, J., Padin, C., & Ferro, C. (2014). Sustainable business models. *Baltic Journal of Management*, *9*(3), 357–380.

[CR45] Homayouni Z, Pishvaee MS, Jahani H, Ivanov D (2021). A robust-heuristic optimization approach to a green supply chain design with consideration of assorted vehicle types and carbon policies under uncertainty. Annals of Operations Research.

[CR46] Houck, B. (2017). *Why some restaurants are cutting ties with mobile ordering apps.* Eater. Retrieved August 16, 2022l, https://www.eater.com/2017/8/29/16214442/restaurant-order-pay-apps-seamless-postmates-uber-eats.

[CR112] Ivanov, D. (2022). Viable supply chain model: integrating agility, resilience and sustainability perspectives—lessons from and thinking beyond the COVID-19 pandemic. *Annals of Operations Research*, *319*(1), 1411–1431. 10.1007/s10479-020-03640-6 PMC7243232 32836614

[CR47] Jawahar N, Satish Pandian G, Gunasekaran A, Subramanian N (2017). An optimization model for sustainability program. Annals of Operations Research.

[CR48] Jha R (2022). A novel hybrid intelligent technique to enhance customer relationship management in online food delivery system. Multimedia Tools and Applications.

[CR49] Jia H, Shen S, RamírezGarcía JA, Shi C (2022). Partner with a third-party delivery service or not? A prediction-and-decision tool for restaurants facing takeout demand surges during a pandemic. Service Science.

[CR50] Joselow, M. (2020). *Delivery vehicles increasingly choke cities with pollution*. Scientific American. Retrieved November 30, 2022, https://www.scientificamerican.com/article/delivery-vehicles-increasingly-choke-cities-with-pollution/

[CR51] Kapoor AP, Vij M (2018). Technology at the dinner table: Ordering food online through mobile apps. Journal of Retailing and Consumer Services.

[CR52] Kathleen, M., & Lo, H.Y. (2022). *Hong Kong social distancing: Malls, delivery platforms seek to turn restaurants’ loss into their gain.* South China Morning Post. Retrieved August 1, 2022, https://www.scmp.com/news/hong-kong/hong-kong-economy/article/3162425/hong-kong-social-distancing-malls-delivery.

[CR53] Kaur P, Dhir A, Talwar S, Ghuman K (2021). The value proposition of food delivery apps from the perspective of theory of consumption value. International Journal of Contemporary Hospitality Management.

[CR54] Kohar A, Jakhar SK (2021). A capacitated multi pickup online food delivery problem with time windows: A branch-and-cut algorithm. Annals of Operations Research.

[CR55] Lang K, Zhao Y (2021). Cloud computing resource scheduling based on improved ANN model takeaway order volume forecast. Journal of Intelligent & Fuzzy Systems.

[CR56] Le Breton C, Galiere S (2022). The role of organizational settings in social learning: An ethnographic focus on food-delivery platform work. Human Relations,.

[CR57] Lee S, Chang HS, Cho M (2022). Applying the sociotechnical systems theory to crowdsourcing food delivery platforms: The perspective of crowdsourced workers. International Journal of Contemporary Hospitality Management.

[CR58] Leung, H. (2022). *‘The price of participation’: For Hong Kong eateries, delivery giants Foodpanda and Deliveroo are a double-edged sword*. Hong Kong Free Press. Retrieved August 30, 2022, https://hongkongfp.com/2022/01/23/the-price-of-participation-for-hong-kong-restaurants-delivery-giants-are-a-double-edged-sword/.

[CR59] Li C, Mirosa M, Bremer P (2020). Review of online food delivery platforms and their impacts on sustainability. Sustainability.

[CR60] Li J, Choi TM, Cheng TCE (2013). Mean variance analysis of fast fashion supply chains with returns policy. IEEE Transactions on Systems, Man, and Cybernetics: Systems.

[CR61] Li L, Chi T, Hao T, Yu T (2018). Customer demand analysis of the electronic commerce supply chain using Big Data. Annals of Operations Research.

[CR62] Lichtenstein, N. (2020). The hidden cost of food delivery. TechCrunch. https://techcrunch.com/2020/03/16/the-hidden-cost-of-food-delivery/.

[CR63] Lin M, Lin S, Ma L, Zhang L (2022). The value of the Physical Internet on the meals-on-wheels delivery system. International Journal of Production Economics.

[CR64] Lin PM, Au WC, Leung VT, Peng KL (2020). Exploring the meaning of work within the sharing economy: A case of food-delivery workers. International Journal of Hospitality Management.

[CR65] Liu GY, Agostinho F, Duan HB, Song GH, Wang XQ, Giannetti BF, Santagata R, Casazza M, Lega M (2020). Environmental impacts characterization of packaging waste generated by urban food delivery services. A big-data analysis in Jing-Jin-Ji region (China). Waste Management.

[CR66] Luo S, Choi TM (2022). E-commerce supply chains with considerations of cyber-security: Should governments play a role?. Production and Operations Management.

[CR67] Marks, G. (2020). *Restaurant owners may not like delivery services–but can they do without them?* The Guardian. Retrieved August 1, 2022, https://www.theguardian.com/business/2020/dec/02/restaurant-owners-delivery-services-grubhub-doordash-fees-pandemic.

[CR68] McLean, H. (2020). *SkipTheDishes to help restaurant partners with 30-day support package*. Dished. Retrieved August 16, 2022, https://dailyhive.com/vancouver/skipthedishes-restaurant-support-package-canada.

[CR69] Mok, D. (2020). *Deliveroo couriers in Hong Kong take protest over new pay policy into third day*. South China Morning Post. Retrieved August 16, 2022, https://www.scmp.com/news/hong-kong/society/article/3086032/deliveroo-couriers-hong-kong-take-protest-over-new-pay.

[CR70] Mok, T. (2021). *Toronto restaurant owner calls out customers scamming delivery apps for free food*. blogTO. Retrieved August 16, 2022, https://www.blogto.com/eat_drink/2021/03/toronto-restaurant-owner-calls-out-customers-scamming-delivery-apps-free-food/.

[CR71] Moncef B, Dupuy MM (2021). Last-mile logistics in the sharing economy: Sustainability paradoxes. International Journal of Physical Distribution & Logistics Management.

[CR72] Muszyński K, Pulignano V, Marà C (2022). Product markets and working conditions on international and regional food delivery platforms: A study in Poland and Italy. European Journal of Industrial Relations.

[CR73] Niu B, Li Q, Mu Z, Chen L, Ji P (2021). Platform logistics or self-logistics? Restaurants’ cooperation with online food-delivery platform considering profitability and sustainability. International Journal of Production Economics.

[CR74] Pandey S, Chawla D, Puri S (2022). Food delivery apps (FDAs) in Asia: An exploratory study across India and the Philippines. British Food Journal.

[CR75] Piasna A, Drahokoupil J (2021). Flexibility unbound: Understanding the heterogeneity of preferences among food delivery platform workers. Socio-Economic Review.

[CR76] Preetha, S., & Iswarya, S. (2019). An analysis of user convenience towards food online order and delivery application (Food app via platforms). *International Journal of Management. Technology and Eng*ineering, pp. 429–433.

[CR77] Puram P, Gurumurthy A, Narmetta M, Mor RS (2022). Last-mile challenges in on-demand food delivery during COVID-19: Understanding the riders' perspective using a grounded theory approach. The International Journal of Logistics Management.

[CR78] Qu, T. (2022). *Chinese directive asking on-demand platforms to lower merchant fees triggers sell off in Meituan shares in Hong Kong*. South China Morning Post. Retrieved January 18, 2023, https://www.scmp.com/tech/policy/article/3167567/chinese-directive-asking-demand-platforms-lower-merchant-fees-triggers?module=inline&pgtype=article

[CR79] Raza A, Asif M, Akram M (2022). Give your hunger a new option: Understanding consumers’ continuous intention to use online food delivery apps (OFDAs) using trust transfer theory. International Journal of Consumer Studies, Published Online,.

[CR80] Schulz SA, Flanigan RL (2016). Developing competitive advantage using the triple bottom line: A conceptual framework. Journal of Business & Industrial Marketing.

[CR81] Seghezzi A, Mangiaracina R (2020). On-demand food delivery: Investigating the economic performances. International Journal of Retail & Distribution Management.

[CR82] Seghezzi A, Winkenbach M, Mangiaracina R (2021). On-demand food delivery: A systematic literature review. The International Journal of Logistics Management.

[CR83] Shah, K. (2020). *Delivery platforms need to give restaurants a break*. Food & Wine. Retrieved August 1, 2022, https://www.foodandwine.com/fwpro/delivery-apps-restaurants-coronavirus-commission.

[CR84] Shanahan G, Smith M (2021). Fair’s fair: Psychological contracts and power in platform work. The International Journal of Human Resource Management.

[CR85] Sherred, K. (2019). *Why “ghost” restaurants are changing the delivery game*. Restaurant Dive. Retrieved November 30, 2022, https://www.restaurantdive.com/news/why-ghost-restaurants-are-changing-the-delivery-game/546624/

[CR86] Sheu JB, Chen YJ (2012). Impact of government financial intervention on competition among green supply chains. International Journal of Production Economics.

[CR87] Sheu JB, Choi TM (2022). Can we work more safely and healthily with robot partners? A human-friendly robot-human coordinated order fulfillment scheme. Production and Operations Management.

[CR88] Sin KY, Lo MC, Mohamad AA (2021). The determinants and barriers of outsourcing third-party online delivery: Perspectives of F&B entrepreneurs in Malaysia. Journal of Asian Finance, Economics and Business.

[CR89] Sinha D, Pandit D (2021). A simulation-based study to determine the negative externalities of hyper-local food delivery. Transportation Research Part D: Transport and Environment.

[CR90] Song M, Du Q, Zhu Q (2017). A theoretical method of environmental performance evaluation in the context of big data. Production Planning & Control.

[CR91] Song M, Fisher R, de Sousa Jabbour ABL, Santibañez Gonzalez ED (2022). Green and sustainable supply chain management in the platform economy. International Journal of Logistics Research and Applications.

[CR92] Spence, A. (2022). *Driver refused to deliver order 12 miles away after customer changed address*. Newsweek. Retrieved August 1, 2022, https://www.newsweek.com/driver-refused-deliver-order-12-miles-away-after-customer-changed-address-1673986.

[CR93] Sun P, Chen JY, Rani U (2021). From flexible labour to ‘sticky labour’: A tracking study of workers in the food-delivery platform economy of China. Work, Employment and Society.

[CR94] Sun Y, Wu Z, Zhu W (2022). When do firms benefit from joint price and lead-time competition?. European Journal of Operational Research.

[CR95] Taylor TA (2018). On-demand service platforms. Manufacturing & Service Operations Management.

[CR96] Tsai PH, Hsiao WH, Chen CJ (2022). Which food delivery platforms are winning the restaurant food delivery wars? Analysis from a consumer perspective. International Journal of Consumer Studies.

[CR97] Ulmer MW, Thomas BW, Campbell AM, Woyak N (2021). The restaurant meal delivery problem: Dynamic pickup and delivery with deadlines and random ready times. Transportation Science.

[CR98] Veen A, Barratt T, Goods C (2020). Platform-capital’s ‘app-etite’ for control: A labour process analysis of food-delivery work in Australia. Work, Employment and Society.

[CR99] Wang HT (2022). Analysis of a tripartite evolutionary game model of food delivery platform supervision and strategy selection. Technology Analysis & Strategic Management.

[CR100] Wang X, Wang L, Wang S, Chen JF, Wu C (2021). An XGBoost-enhanced fast constructive algorithm for food delivery route planning problem. Computers & Industrial Engineering.

[CR101] Wang Z, He SY (2021). Impacts of food accessibility and built environment on on-demand food delivery usage. Transportation Research Part D: Transport and Environment.

[CR102] Yang Y, Liu H, Chen X (2020). COVID-19 and restaurant demand: Early effects of the pandemic and stay-at-home orders. International Journal of Contemporary Hospitality Management.

[CR103] Yao X, Cheng Y, Zhou L, Song M (2020). Green efficiency performance analysis of the logistics industry in China: Based on a kind of machine learning methods. Annals of Operations Research.

[CR104] Yen YS (2022). Channel integration affects usage intention in food delivery platform services: The mediating effect of perceived value. Asia Pacific Journal of Marketing and Logistics.

[CR105] Yeo SF, Tan CL, Teo SL, Tan KH (2021). The role of food apps servitization on repurchase intention: A study of FoodPanda. International Journal of Production Economics.

[CR106] Young, A. (2021). *Most gig economy workers have been injured at work–and tool unpaid time off*. Mirror. Retrieved November 30, 2022, https://www.mirror.co.uk/money/jobs/gig-economy-workers-injured-work-25587129.

[CR107] Zhang T, Choi TM, Zhu X (2018). Optimal green product’s pricing and level of sustainability in supply chains: Effects of information and coordination. Annals of Operations Research.

[CR108] Zhang W, Dai Y, Tian L (2022). Impact of capacity flexibility on service product line design. Annals of Operations Research.

[CR109] Zheng J, Wang L, Wang L, Wang S, Chen JF, Wang X (2022). Solving stochastic online food delivery problem via iterated greedy algorithm with decomposition-based strategy. IEEE Transactions on Systems, Man, and Cybernetics: Systems.

[CR110] Zheng Q, Zhan J, Feng X (2022). Working safety and workloads of Chinese delivery riders: The role of work pressure. International Journal of Occupational Safety and Ergonomics.

[CR111] Zhu X, Yang C, Liu K, Zhang R, Jiang Q (2022). Cooperation and decision making in a two-sided market motivated by the externality of a third-party social media platform. Annals of Operations Research.

